# Is a Mesh Really a Mess: A Self-Tailored Polypropylene Mesh as a Retropubic Tension-Free Vaginal Tape Under Local Anesthesia in a Tertiary Healthcare Center Experience in India

**DOI:** 10.7759/cureus.43957

**Published:** 2023-08-23

**Authors:** Sidhartha Kalra, Sovan Hota, Atanu Kumar Pal, Lalgudi N Dorairajan, Sreerag Kodakkattil Sreenivasan, Vishal Narkhede

**Affiliations:** 1 Urology and Renal Transplantation, Jawaharlal Institute of Postgraduate Medical Education and Research, Pondicherry, IND

**Keywords:** daycare surgery, sling surgery, self-tailored polypropylene mesh, tension-free vaginal tape, stress urinary incontinence

## Abstract

Objective

The primary objective of this study was to evaluate the feasibility of performing retropubic mid-urethral transvaginal tape (TVT) with self-tailored ordinary polypropylene mesh (STOM) under sedation and local anesthesia in female patients with stress urinary incontinence (SUI). The second objective was to evaluate perioperative and immediate postoperative complications, success rates, and cost-effectivity.

Materials and methods

Forty-two subjects treated for stress urinary incontinence using STOM under local anesthesia were reviewed. Pre- and postoperative evaluation included assessment of valid questionnaires such as the urogenital distress inventory (UDI) and medical, epidemiologic, and social aspects of aging incontinence questionnaire (MESA), uroflowmetry in all cases, and urodynamics in some instances. Success rates and complications were compared with published studies.

Results

The mean operating time was 27 mins, and the median visual analog scale (VAS) score at 12 hours was three. Postoperative MESA and UDI scores had significant improvement. The mean hospital stay was 18 hours. Mean preoperative and postoperative Q max had no significant difference. With a mean follow-up of 27.32±3.29 months, the cure was seen in 38 patients (90.4%), improvement in three patients (0.07%), and failure in one patient (0.02%). Mesh-related complications (extrusion) occurred in one patient. The sling cost was reduced from approximately $500 (Gynecare TVT sling; Ethicon Inc., Somerville, NJ, USA) to $12.44 (Ethicon 15 x 7.6 cm Prolene (polypropylene mesh); Ethicon Inc., Somerville, NJ, USA) in our study.

Conclusion

Performing TVT with STOM under sedation and local anesthesia as a daycare procedure was feasible and cost-effective, has a high success rate, and was associated with minimal complications. It should be considered in developing countries with vast patient burdens, such as India.

## Introduction

The concept of retropubic mid-urethral transvaginal tape (TVT) using synthetic mesh as a management strategy for stress urinary incontinence (SUI) has been popularised since its introduction in 1996. In 2011, the US Food and Drug Administration (FDA) issued a safety alert for the use of transvaginal mesh implants to treat female prolapse as complications such as infection, chronic pain, dyspareunia, vaginal erosion, shrinkage, and erosion into other organs were reported from numerous studies [1]. Though FDA public health notifications did not specifically warn against the use of synthetic mesh for SUI, there was evidence in the literature about the immediate decreased use of synthetic mesh slings for treating SUI at many academic tertiary care centers [2]. A consensus group meeting convened by the European Association of Urology (EAU) and the European Urogynecological Association concluded that Synthetic slings could be safely used in the surgical treatment of SUI in both male and female patients [3]. A recent meta-analysis showed a success rate of 89.1% for retropubic mid-urethral sling (i.e., TVT) in women with SUI [4]. 

The tension-free vaginal tape procedure by a sub-urethral retropubic sling made out of mesh (TVT) invented by Ulmsten et al. has become the gold standard [5]. This procedure requires special needles or passers and expensive prefabricated slings. In developing countries, the cost factor of these commercial kits is prohibitive for most SUI patients. Already a few studies have described the successful use of ordinary polypropylene mesh and specifically designed needles in the mid-urethral tension-free vaginal trans-obturator tape (TVT-O) and pubovaginal sling procedure [6-10]. Although commonly used, no studies have described its use in the retropubic mid-urethral TVT sling procedure.

Local anesthesia (LA) is a good option for placing TVT. A prospective randomized trial of sedation with local anesthesia versus general anesthesia (GA) for the TVT procedure documented shorter recovery time, lower pain scale, and no difference in the rate of complications in the LA group [11].

We describe our experience using self-tailored ordinary polypropylene mesh (STOM) and helical needles in mid-urethral TVT sling procedures under local anesthesia in 42 patients treated in our tertiary care center. We evaluated the clinico-demographic data, mean operating time, perioperative complications, hospital stay, improvement, failure rates, and cost-effectiveness of this procedure in this study.

## Materials and methods

Patient selection 

From January 2018 to January 2021, 48 patients underwent retropubic mid-urethral TVT at our tertiary care institute. In four patients, the surgical procedure was done under spinal anesthesia and associated procedures such as anterior or/and posterior colporrhaphy. The procedure was done with local anesthesia in the rest of the patients. Two patients lost to follow-up after 10 months. The remaining 42 patients were included in this study. This study is a retrospective analysis of data collected from an IRB-approved database (JIP/IEC/2020/089). A single well-experienced surgeon performed all the procedures. Consent from study participants and the institutional ethics committee approval was taken. Inclusion criteria included all women with stress-predominant urinary incontinence, a history of symptoms for at least three months with failed conservative management, postvoid residual urine (PVRU) < 100 ml, and a negative urine culture. The perioperative data included urology and gynecological history, pelvic examinations, validated questionnaires such as the urogenital distress inventory (UDI); medical, epidemiologic, and social aspects of aging incontinence questionnaire (MESA); cough stress test; uroflowmetry (Q max); and visual analog scale (VAS) score for pain. A urodynamic study with an assessment of detrusor pressure, detrusor overactivity, and abdominal leak point pressure (ALPP) was done for cases with mixed urinary incontinence and previous failed incontinence surgery. ALPP of less than 60 cm H2O indicated intrinsic sphincter deficiency. The MESA is a self-reported questionnaire with nine questions on stress incontinence and six on urge incontinence. Both MESA and UDI scores were used to identify predominant stress incontinence.

Exclusion criteria were urgency-predominant mixed urinary incontinence, associated neurological diseases and lesions, post-radiation cases, impaired bladder compliance, and third-degree urogenital prolapse (Baden and Walker classification).

Surgical instruments and self-tailored ordinary polypropylene mesh (STOM)

**Figure 1:**
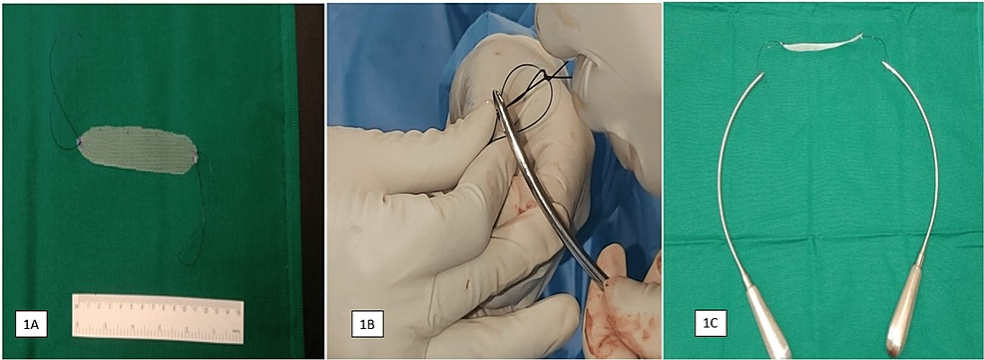
1A: Self-tailored ordinary polypropylene mesh (STOM) of size 11x1.5 cm; 1B: 1-0 Prolene suture anchored to the ends of polypropylene mesh being fixed to the fenestrated end of the re-sterilizable modified transvaginal tape (TVT) needle; 1C: Self-tailored ordinary polypropylene mesh (STOM) fixed to TVT needle.

This modified TVT needle is made of specifically designed stainless steel and comes in pairs for both the left and right sides. It is re-sterilizable and comprises a semi-circular-shaped section and a handle. It is fenestrated at the tip, allowing the insertion of polypropylene sutures attached intraoperatively to both ends of the polypropylene mesh strip (Figure 1).

The commercially available, 15 × 7.6 cm, nonabsorbable monofilament polypropylene mesh (Prolene®; Ethicon Inc., Somerville, NJ, USA) (the same mesh used in mesh hernioplasty) was tailored to make an 11 x 1.5 cm size tape. Polypropylene suture (1-0) was anchored to each end of the polypropylene tape in a figure-of-eight fashion. The polypropylene suture was inserted into the "eye" at the end of the helical needle (Figure 1).

Local anesthesia

**Figure 2:**
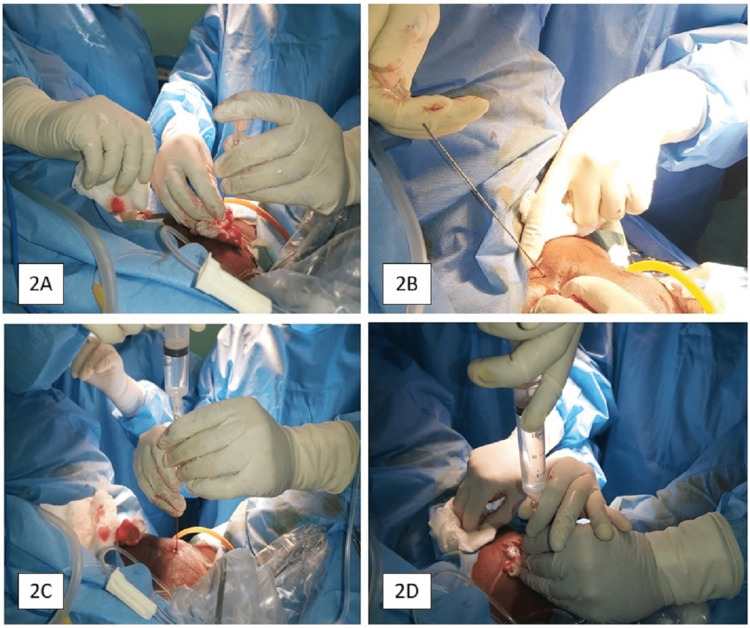
Techniques of local anesthesia injection. 2A: Initial vertical direction of the needle till it pierces the rectus fascia; 2B: Oblique direction of the needle till it pierces the endopelvic fascia; 2C and 2D: Local anesthesia is being injected.

This procedure was carried out with local anesthesia in the lithotomy position. Inj. Ketorolac of 0.5 mg/kg and midazolam of 0.2 mg/kg were used for analgesia and sedation, respectively. The mixture of 15 mL of 2% lidocaine with 1:100,000 epinephrine, 10 mL of 8.4% NaHCO3, and 10 mL of normal saline was used to make the solution for tumescent anesthesia. Additionally, 5 mL was injected into each proposed line of incision on either side of the suprapubic region; 10 mL was injected on each side after going deep into the rectus fascia; and 2% xylocaine gel was applied to the vaginal mucosa 30 minutes before the procedure. A paired 1 cm-length mark was made 2 cm from the midline over the suprapubic region. The local anesthetic mixture was injected through the marked suprapubic area with a long Chiba needle of 22G (Figure 2).

The needle was initially inserted vertically till it pierced the rectus fascia and then directed obliquely. At the same time, it traversed on the back of the pubic bone at the space of Retzius till it pierced the endopelvic fascia and the typical "give away" sensation is felt. Then, 5 mL of the mixture was injected at the submucosal level (hydro-dissection) to elevate the vaginal mucosa easing the dissection.

Surgical procedure 

Third-generation cephalosporin was injected at the beginning and repeated eight and 16 hours after the procedure. The STOM was also made during the same time. Eighteen Fr Foley catheter was inserted into the bladder to drain urine completely. A 2-cm-long vertical incision was made 1 cm below the urethral meatus. The incision was deepened till the periurethral fascia with care taken so that it did not encroach upon the urethra. Subsequently, dissection was carried along laterally toward the inferior ramus of the pubis to reach the endopelvic fascia and then repeated on the opposite side. While the catheterized urethra was deflected away with the help of a catheter guide, a modified helical passer, along with a STOM knotted to it, entered through the vaginal incision. Staying close to the bone, the endopelvic fascia was pierced with the help of two hands for better maneuvering. The needle tip was directed away from the bladder on the back of the pubic bone by angling the needle toward the surgeon's opposite shoulder. The last resistance felt was the rectus fascia. The same procedure was repeated on the opposite side.

The tip of the needles exited at the skin level at the marked suprapubic region. Cystoscopy was done with the 70-degree lens to exclude bladder injury. Inspection of the urethra was done while withdrawing the cystoscope. Then, under vision, the needles were slowly withdrawn by a backward rotational movement to come out through the vaginal incision. Kelly clamp/forceps were used as a spacer between the urethra and the STOM to place the sling in a tension-free manner, and catheterization was done. Both ends of the tape were trimmed. Vaginal and abdominal incisions were closed with interrupted Vicryl 3-0 sutures.

The urethral catheter and vaginal pack were removed after six hours. If the voiding trial was unsuccessful, the patient was discharged with an indwelling catheter for two days. Patients with successful voiding trials were discharged on the same day. Abstinence from sexual intercourse for a month and avoidance of strenuous activity for at least six weeks were advised. Follow-up was done after two weeks, at two months, and then every six months.

Evaluation

The demographics of patients, perioperative data and postoperative VAS score, UDI, and MESA score were reviewed. Follow-up data were checked for any complications. The patients were requested to come to assess the outcome of surgery through telephonic communication. Responding patients were evaluated with a cough stress test, UDI, and MESA score. Repeat uroflowmetry (Q max) and urodynamic studies were taken up in case of persistent or new voiding symptoms.

Surgical outcomes were evaluated by the cough stress test with a comfortably full bladder as "cured," "improved," or "failed." Patients were considered "cured" of SUI if they had a negative cough stress test result and did not report urine leakage during stress. Patients were considered "improved" if they did not leak on the cough stress test but may have had occasional urine leakage during stress; this leakage did not influence their daily activities or require further treatment. Patients who did not meet these criteria were considered to have "failed" treatment.

## Results

A total of 42 patients underwent retropubic mid-urethral TVT under local anesthesia. The mean age, body mass index (BMI), and duration of symptoms were 42.15±6.66 years, 23.4±2.67 kg/m^2^, and 6±1.3 years, respectively. The mean operating time and blood losses were 27±5.7 minutes and 35±12 mL, respectively (Table 1).

**Table 1:** Clinico-demographic and perioperative data of the 42 patients. SD: Standard Deviation; BMI: Body Mass Index; SUI: Stress Urinary Incontinence; VAS: Visual Analog Scale

Parameters	Value
Age (in years), mean ±SD (range)	42.15±6.66
BMI (kilogram per square meter) mean ±SD (range)	23.4 ± 2.67
Parity, median (range)	2 (0-7)
Vaginal deliveries, median (range)	3 (0-7)
Premenopausal patients	15/42
Post-menopausal patients	27/42
SUI duration (in years) median (range)	6 (1-15)
Operative time (in minutes), median (range)	27 (21-42)
Blood loss (in ml), median (range)	35 (23-41)
Postoperative VAS score (median)	
At 1st hour	7 (4-9)
At 12th hours	3 (0-6)
At 24th hour	2 (0-5)
Request for antiemetics (number of patients)	3
Length of stay (in hours), median (range)	18 (8-24)
Follow-up (in months), mean± SD (range)	27.32±3.29 (17-31)

The mean follow-up period was 27.32±3.29 months. Thirty-eight (90.4%) women reported complete cures, and 3 (7.14%) were signifi­cantly improved. The surgery failed in 1 (2.38%) patient (Table 2).

**Table 2:** Postoperative complications.

Complications	Number of patients
Bladder perforations	00
De novo urgency	05 (11%)
Vaginal discharge	03 (7.14%)
Dyspareunia	02 (4.76%)
Mesh erosion	01 (2.38%)
Urinary tract infection (UTI)	02 (4.76%)
Groin pain	00
Retention of urine	00
Felt suture	02 (4.76%)
failure	01 (2.38%)

The mean VAS score at 12 hours was mild (three). An additional dose of non-opioid analgesia (Ketorolac) was required only in five patients after 12 hours in the postoperative period. Postoperative MESA and UDI scores had significant improvement (Table 3). Mean preoperative and postoperative Q max had no significant difference (Table 3).

**Table 3:** Preoperative and postoperative comparison. UDI: Urogenital Distress Inventory MESA: Medical, Epidemiologic, and Social Aspects of Aging Incontinence Questionnaire Q max: Maximum Flow Rate in Uroflowmetry.

Parameters	Preoperative score	Postoperative score (2 months)
MESA	23.2±3.6	2.25±1.4
UDI-6	33.3±2.7	12.5±2.3
Q max (ml/second)	27.8±6.85	27.3±4.49

Per urethral, Foley's catheter was kept for six hours in 34 patients and 24 hours in six patients. The voiding trial failed 24 hours in two patients, so they were sent home after teaching self-intermittent catheterization. The repeat voiding trial was successful after two days in both. However, one presented with bothering urgency symptoms and PVRU of 150 mL with normal Qmax, which gradually improved over six weeks.

Mesh excision was required in case of extrusion after six months of surgery in a diabetic patient. Postoperative de novo urgency was observed in five patients for three months and managed with beta-3 agonists and anticholinergic agents. Postoperative serous discharge was evident in three patients even after one week of surgery, which was treated with oral antibiotics according to culture sensitivity and local antiseptics. Two patients complained of mild discomfort because they could feel sutures subcutaneously over the abdomen. No bladder injuries were reported, which was confirmed with a 70-degree scope. UTI and dyspareunia were reported in two patients each, respectively (Table 2). UTI was treated with antibiotics as per urine culture and sensitivity.

## Discussion

SUI is a devastating condition that affects many females. The basic idea of all sling and tape procedures is the placement of a strip, either organic or synthetic material, under the bladder neck and urethra, producing urethral support without compression during a sudden increase in intra-abdominal pressure. Ulmsten et al. described the procedure of TVT based on the urethral closure mechanism in women [5].

Nygaard et al. reported that SUI affects 50%-83% of women over 65 years of age [12]. Singh et al. found a prevalence of incontinence in 21.8% of women in their hospital-based cross-sectional study of 3,000 women in India. Of them, 73.8% were found to have stress incontinence [13]. Fewer of them seek treatment as SUI is associated with social stigma, and management depends mainly on the quality of life and economic status of the patients. Commercial TVT kits are costly and not widely affordable in developing countries. We used traditional polypropylene mesh of hernias repair as the material for the sling. It has good memory, tissue integration, minimum shrinkage, and adhesion potential and is easy to use. The mesh is characterized by relatively wide pores, which allow the circulation of macrophages, fibroblasts, deposition of collagen, and angiogenesis to promote the good healing of the vaginal wound with low vaginal and urethral erosion rates [11].

Various studies have already documented the reasonable safety and efficacy of ordinary polypropylene mesh in sling procedures. ElSheemy et al. evaluated an ordinary polypropylene mesh's long-term safety and effectiveness in the TVT-O procedure [7]. The complications were vaginal discharge (6%), dyspareunia (1%), groin pain (20%), UTI (3%), and obstructive symptoms (1%). They had no cases of erosions or de novo urgency. Of the 59 women, 91% were cured, 5% improved, and 3% failed. Shah et al. evaluated the five-year outcome of a self-tailored mesh as a pubovaginal sling [8]. Of their 49 patients, 40 (82%) were dry, and two (4%) improved. De novo urgency and urgency urinary incontinence (UUI) were reported in one patient each. Three patients (8%) had recurrent SUI, while prolonged retention developed with subsequent urethrolysis required in two (4%). None of the patients had an infection, failed to heal, or erosion of the synthetic slings. Thus, previous studies of self-tailored mesh, either as pubovaginal sling or TVT-O, have a comparable outcome with commercial kits.

The prevalence of SUI in middle-aged Indian women is around 16%. Sandhu et al. obtained the objective cure rate of 85% at the end of one year in their study conducted in India. The improvement in patient satisfaction in both UDI-6 and incontinence impact questionnaire-7 (IIQ-7) was statistically significant [14]. In our research, we have seen statistically significant improvement in UDI-6 and MESA scores.

LA promotes early ambulation, thereby reducing the hospital stay and eventually cost. Unlike GA, extensive preoperative evaluations are not required. A prospective randomized trial by Araco et al. documented a lower VAS scale, reduced recovery room duration, discharge on the same day in more patients, and no difference in the rate of complications in the LA group compared to the GA group for the patients who underwent tension-free vaginal tape Secur hammock procedure and discharged at least one day after surgery [11]. A retrospective study by Rajamaheswari et al. on 46 patients who underwent TVT under local anesthesia showed an 88% cure rate and significant improvement in 7.4% of the patients and failure in 3.7% of patients [15]. The mean operative duration was 29 minutes. Bladder perforation was observed in six patients. A recent study supported the idea of using LA for TVT placement in SUI, where 302 patients had subjective and objective cure rates were 92% and 93%, respectively. The 12-month sling section rate and long-term self-catheterization rate were only 1% and 0.3%, respectively [16].

Our study recorded the cure rate at 90.4%. Failure was observed in one patient with SUI with a severe intrinsic sphincter deficiency (ISD) component. The success rate was similar to the results of other studies on TVT as per literature (89-91%). The operative duration was 27 min, comparable to other studies of TVT under local/regional anesthesia. In their research, Schmitt et al. documented that the objective and subjective cure rates were 93% and 92%, respectively [16]. We also had similar outcomes. The STOM was created while the operative procedure was going on. Hence, it did not lead to an increase in the duration of surgery. Postoperatively, the duration of the per-urethral catheter (PUC) in situ was six hours in most patients, and they were discharged on the same day. Most of the patients underwent TVT as daycare surgery, which is a boon to our tertiary care center with a huge waiting list of patients for admission. Moreover, affordable daycare treatment improves women's mental health, as per a study conducted in rural India [17]. Complication rates (Table 2) were comparable to the previous studies of commercial kits under LA/GA [11] and self-tailored mesh under GA [7]. The early postoperative voiding difficulty was reported in two patients; however, both had a complete resolution over six weeks. The reported rate of voiding difficulty in literature after TVT procedures ranged from 2.5% to 8.5% [18]. Bladder perforations (0%), urgency (11%), vaginal discharge (7.14%), dyspareunia (4.76%), mesh erosion (2.38%), and UTI (4.76%) rates were similar to a series of 446 patients [19].

The use of nonabsorbable synthetic material as a sling has been questioned due to reports of erosion and infection. As the U.S. FDA advisory committee suggested in August 2021, transvaginal pelvic organ prolapse (POP) mesh does not have a favorable benefit-risk profile [20]. The British Association of Urological Surgeons (BAUS) raised significant concern over using synthetic polypropylene mesh in SUI or POP in the statement released in April 2019 due to the lack of quality long-term data [21]. Efforts made to manufacture novel biomaterials are still under experiments [22]. Contrary to these concerns regarding complications, our study of self-tailored mesh with a mean follow-up of 27.32±3.29 months showed a cure rate of 90.4%, mesh erosion in only 0.02% of patients, and urinary retention in none. Surgeons’ experiences, as well as technicality, played a significant part. We believe that synthetic mesh does not create a mess. Instead, it benefits the patients with SUI in the long term.

Our study is limited by the relatively few patients and the short follow-up (27.32+3.29 months). As a descriptive series, there was no control group, and our results were compared with other published studies. The study's strengths are that a uniform assessment of SUI was done using standardized questionnaires in all patients, in addition to using urodynamics for an objective evaluation in some instances.

In tertiary centers with a heavy burden of surgical procedures, a decrease in total operation theatre time has significant value. The use of local anesthesia led to a reduction in theatre time in our study. Most of the patients were discharged the same day after a successful voiding trial. Our technique was also economical as it reduced the material cost approximately from $500 (Gynecare TVT sling; Ethicon Inc., Somerville, NJ, USA) to $12.44 (Ethicon 15 x 7.6 cm Prolene mesh; Ethicon Inc., Somerville, NJ, USA). Furthermore, our modified helical passers were used for all STOM cases as they are re-sterilizable instruments. Thus, they did not add to the treatment expenses. These advantages are significant in developing countries due to limited healthcare resources.

## Conclusions

Retropubic mid-urethral TVT with a STOM under local anesthesia is a simple, safe, effective, reproducible, and economical surgical procedure for treating SUI. This should be considered a low-cost alternative to commercial kits for treating female SUI, mainly for public health systems with limited financial resources. However, randomized controlled studies with a large cohort of patients and long-term follow-ups are needed to confirm our results concerning safety and effectiveness.
